# Rate of Force Development During a Handgrip Task Is Correlated with the Post-Impact Ball Speed of the Flat Serve

**DOI:** 10.3390/sports12110292

**Published:** 2024-10-24

**Authors:** Károly Dobos, Dario Novak, János Péter Tóth, Csaba Ökrös

**Affiliations:** 1 Saint John Apostle Catholic Elementary School and Kindergarten, 1043 Budapest, Hungary; 2 Department of Sport Games, Hungarian University of Sports Science, 1123 Budapest, Hungary; toth.peter.janos96@gmail.com (J.P.T.); okros.csaba@tf.hu (C.Ö.); 3 Faculty of Kinesiology, University of Zagreb, 10000 Zagreb, Croatia; dario.novak@kif.unizg.hr; 4 Institute for Anthropological Research, 10000 Zagreb, Croatia

**Keywords:** ball speed, contact point, handgrip, flat serve, rate of force development of dominant arm

## Abstract

The aim of the present research was to test the hypothesis that rate of force development (RFD) during a handgrip task of the dominant arm in three different positions is associated with maximal post-impact ball speed of flat serve (PIBS). Altogether 23 elite junior boys (aged 14.84 ± 2.47 years; weight 59.51 ± 13.83 kg; height 170.47 ± 16.34 cm) tennis players participated in the study. To assess the maximal voluntary contraction (MVC) and RFD during the task, four handgrip tests and a serve test were applied to estimate PIBS. Spearman’s rank correlation showed a significant positive correlation between RFD of dominant arm in each three position and PIBS (r = 0.82–0.86; *p* < 0.001). A very large, significantly positive correlation was also found between MVC of the dominant arm and PIBS (r = 0.88; *p* < 0.01). The result of the present study indicated that rapid force generation of muscles in the forearm and wrist may probably play a role in the formation of a stable contact point, and it is in connection with PIBS. It is in contrast to the slow maximal force exertion that much rather characterizes the general strength state of players. Therefore, measurement of the RFD during a handgrip task is suggested in the testing session of the flat serve of junior tennis players.

## 1. Introduction

Technical elements of tennis involve numerous forearm movements. Forearm bones are covered by a double-layer of extensor and flexor muscles, both on the surface and in the deep layers. During the execution of tennis strokes, these extensor and flexor muscles contract forcefully and rapidly immediately before and at the ball contact [1,2,3,4,5,6], while a rapid relaxation occurs after the ball contact [7].

Forearm muscles play a huge role in the stabilization of the wrist, providing a stable contact point at the stroke, as well as in preventing injuries [5,8]. Furthermore, in tennis, the ball is forwarded in an indirect way with a racket. The time of contact between the racket and the ball is extremely short [5,9], during which the player has to impart the proper direction, trajectory, and force impact on the ball. That is why the ability of the player to handle the racket well is of utmost importance, which may be predicted by measuring the handgrip force.

Earlier researchers [10,11,12,13,14,15] formulated a hypothesis that handgrip of the dominant arm with maximal voluntary contraction (MVC) influences the maximal post-impact ball speed of the flat serve (PIBS), but contradicting results were found. It is possible, however, that other mechanical variables of muscles contribute to PIBS.

Several studies have proven the controlling role of the proper handgrip force in decreasing the vibration of the elbow [7,16,17,18,19]. Knudson and White [3] have demonstrated that the level of force exertion of the racket grip shows an increased change during the swing and becomes consistent (stable) only about 0.1 s prior to ball contact. Thus, players are recommended to perform the swing with low force application and rapidly increase force shortly before the actual stroke is executed [5,20]. In contrast to slow MVC, the rate of force development (RFD) of the dominant arm would probably predict PIBS. Our hypothesis also derives from previous evidence that during flat serve, the applied handgrip force is about 31–44% of the MVC [14]. However, less information is available on this special topic for junior tennis players.

Therefore, the aim of the present research was to test the hypothesis that RFD during a handgrip task performed in different positions with the dominant arm of elite junior tennis players is associated with the PIBS and to examine the correlation of MVC of the dominant hand in the basic position with the PIBS.

## 2. Materials and Methods

### 2.1. Participants

In the study, 23 elite junior boy tennis players (chronological age: 14.84 ± 2.47 years; body weight: 59.51 ± 13.83 kg; body height: 170.47 ± 16.3 cm) were involved. Sampling was carried out with a professional sampling method in order to select those tennis players who possessed the correct serve technique at the highest level in their age categories. In other words, the serving movement of the selected players had to contain exactly the most relevant key movements or check points (preparation and stance, backswing, toss, trophy position, forward swing to impact, follow through) that play a role in the execution of the appropriate serve technique. Key movement of serve was checked according to instructions by Elliott et al. [20]. Further criteria were that the player had to have 2 to 7 years of tennis competition experience in tournaments, and had to play 30–60 matches per year.

Within the sample, 20 participants were right-handed, 3 left-handed, and all of them had a two-handed backhand.

All players were among the top 30 on the Hungarian under 12, 14, 16, and 18 national ranking list in their age categories. Furthermore, 26% of the players had a European Tennis Association (ETA) or International Tennis Federation (ITF) ranking.

The players had no signs of injury in the upper extremities at least 2 months before the testing sessions, and no injuries occurred during the tests. Players were familiarized with testing protocol one week before the testing sessions; they learned how to use the handgrip dynamometer to execute handgrip tasks and tennis serve tests. Before any testing session, players were informed in both oral and written form about the testing procedures. All of the players had medical screening and received declarations of consent from their parents. Furthermore, the study met the guidelines of ethical standards for sports medicine [21] and was approved by the ethical committee of the Public Health Division of Budapest Government Office.

### 2.2. Instruments

To measure the MVC and RFD of the dominant arm, a Dyna-8 FMS dynamometer was used. Software-hardware system of instrument specially designed for isometric measurement and S type load cell (Jiangxi SOP Precision Intelligent Manufacturing Technology Co., Ltd., Ganzhau, China, Model number: SOPLY-107) transducer/sensor was used for data collection and processing. The specification of the instrument is as follows: linearity and hysteresis ±1.5%, range of force measurement 20–1200 N, sampling frequency 300 HZ, and capacity 0–200 kg. Any other information about this dynamometer was described in this article [22].

The PIBS was measured with the Stalker ATS II (Applied Concept, Inc., Dallas, TX, USA) radar gun (with ±3 km/h of accuracy and operating frequency: 34.7 GHz [Ka-Band] ± 50 MHz). During the test, the players used their own tennis rackets with individually adjusted vertical and horizontal string tension matching their own playing style in order to aid the movement execution at the highest level and hit the target area. Furthermore, brand new 53–56 g and 6.5 diameter “Slazenger Ultra Vis” balls were used that met international standards. Finally, before each testing session, the handgrip dynamometer and the radar gun were calibrated in accordance with the manufacturer’s specifications.

### 2.3. Examined Variables and Procedures

Present research was conducted to assess the special motor skills, with a focus on the RFD of the dominant arm and to estimate the MVC of the dominant arm as well as PIBS. Five tests: handgrip test 1 (HGT1)—to assess the MVC of the dominant arm in the basic position; handgrip test 2 (HGT2)—to assess the RFD of the dominant arm in the basic position; handgrip test 3 (HGT3)—to assess the RFD of the dominant arm in the shoulder position; handgrip test 4 (HGT4)—to assess the RFD of the dominant arm in the flat serve position; serve test (ST)—to assess the PIBS were used to reach the aim. After these tests, an analysis was carried out to identify the correlation between HGT assessments and the PIBS in order to test the hypothesis.

**Figure 1:**
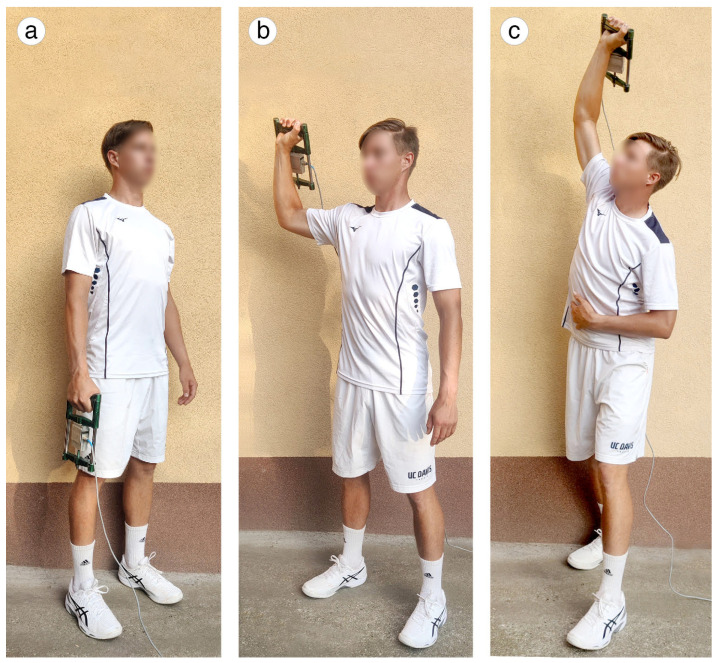
Three different positions in the handgrip measurement. (self-produced figure). Legend: (**a**)—basic position; (**b**)—shoulder position; (**c**)—flat serve position.

HGT1 (N): During the test, the tennis player was standing in a shoulder-width straddle position, holding the dynamometer with a straight arm (elbow fully extended), forearm and wrist in natural position beside the body (Figure 1a). The grip of the device was in the palm with fingers at 90° flexion of the proximal and distal interphalangeal joints with the thumb in 90° abduction. In this test, the handgrip was performed slowly with maximal effort, and the contraction was sustained for 5 s, until the command “stop” was given. MVC was recorded in newton (N) using a special instrument. The test was performed only with the dominant arm, and two trials were allowed. The best result was used for the statistical analysis.

HGT2 (N/s): Position of the tennis player and grip of the instrument were completely identical to those described in the MVC test (Figure 1a). The player was instructed to perform the handgrip as fast as possible on command during the given time limit (0.5–1.5 s). RFD was recorded in N/s. The best result was used for calculation.

HGT3 (N/s): The tennis player was standing in a shoulder-width straddle position while dominant arm was abducted into 90° in relation to the trunk with elbow flexed at 90°, forearm and wrist in natural position at head height (Figure 1b). The player also had to perform the handgrip as fast as possible on command during the given time limit (0.5–1.5 s). RFD was recorded in N/s. The best result was documented for data analysis.

HGT4 (N/s): The tennis player was in a flat serve position. The rear leg was fully extended, the front one slightly bent, the trunk tilted above horizontal, the arm abducted in relation to the trunk, the elbow slightly bent, the wrist bent into the radial direction, and in a slightly extended position above the head (Figure 1c). The player was instructed to perform the handgrip as fast as possible on command during the given time limit (0.5–1.5 s). RFD was recorded in N/s. The best result was considered during data analysis.

**Figure 2:**
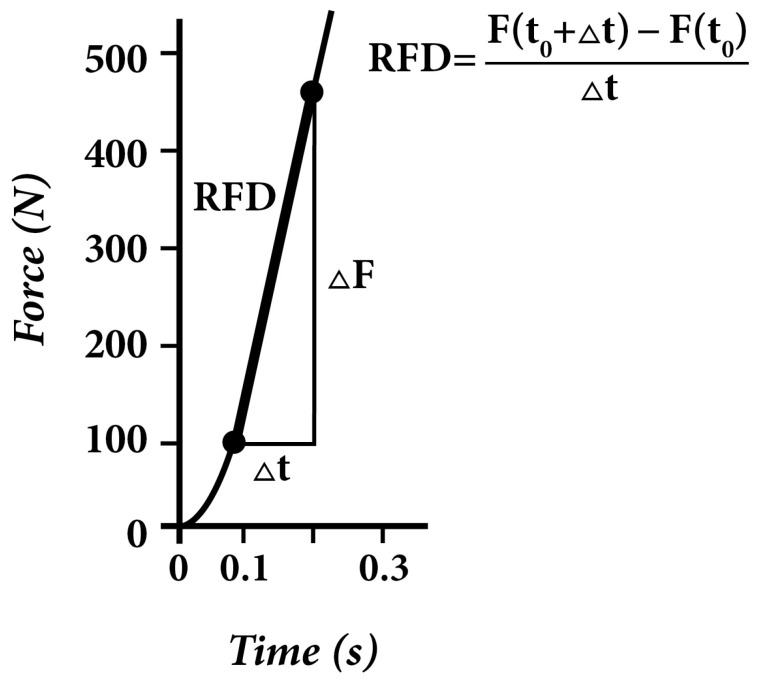
Handgrip force-time curve of junior tennis player. (self-produced figure). The original record shows the rate of force development (*N*) exerted by the muscles of the forearm and wrist (handgrip) of a junior tennis player as a function of time. The change in force (delta *N*) per time (s) at the section of the upward curve where the steepness is maximal indicates the rate of force development during a handgrip task (*N*/s), which is calculated on the equation seen in the figure. Legend: RFD—Rate of force development.

The RFD of handgrip measurement was also conducted only with the dominant arm, and two trials were executed in each position. The RFD calculation was based on the equation: the change in force (delta N) per time (s) at the section of the upward curve where the steepness indicated the maximum (Figure 2).

ST v (Km/h): In the test, the tennis player stood behind the baseline and served from the deuce (right-handed player) or “advanced” (left-handed player) side of the court. He executed 8 flat serves into the 180 × 180 cm target, located in the serve area of the tennis court in the corner nearest to the respective T-line. The player was instructed to execute a flat serve with maximal speed. Only the correctly executed flat serves and the post-impact speeds of those balls landing within the target area were measured. The radar instrument measuring the PIBS was located in the centre, 4 m behind the baseline, at a height covering the contact point of the serve. PIBS was measured in Km/h. The best results of the correct trials were considered for later analysis.

The research was carried out in two stages: (1) evaluating the players’ handgrip force and (2) assessing the PIBS. All measurements were taken within a single day. Before the testing session, the chronological age, body weight, and body height were measured. Body weight was measured with a Beurer digital personal scale with an accuracy of 0.1 kg, and body height was measured with a Sieber-Hegner anthropometer with a millimetre accuracy. Before the HGT, all players participated in a standardized warm-up, including arm circles into several directions, forearm supinations and pronation, wrist flexion and extensions, and general mobilization. Furthermore, before the ST execution, all participants followed a specific warm-up protocol consisting of 5 min dynamic shoulder movements and 20 slow serves. First, the HGTs were performed immediately after warming up, while the measurement of the PIBS was carried out at the end of the measurement. The tests were conducted in the predetermined order described (HGT 1, 2, 3, 4 and ST) in the previous section. Two-minute intervals were allowed between the HGTs and 1 min between the trials to avoid fatigue. The rest time between the HGT and ST had to be 10 min. Finally, at the SS, 25 s rest periods were given between each serve. Feedback (see specific variables in the test descriptions) was given to players about their actual performance during testing.

### 2.4. Statistical Analyses

The basic statistical data were described as a mean ± standard deviation and minimum, maximum result (Table 1). Distribution of the data was analyzed, during which the Shapiro–Wilk-W test was applied. Test reliability was determined by calculating the intra-class correlation coefficients (ICC) for each variable with Spearman–Brown formula of calculation. The ICC values above 0.75 were accepted as reliable in the test [23,24]. Correlations between MVC in the basic position and RFD of the dominant arm in three different positions, and the PIBS were computed with Spearman’s rank correlation methods. The magnitude of correlation was classified according to Hopkins [25] (trivial = 0–0.1; small = 0.1–0.3; moderate = 0.3–0.5; large = 0.5–0.7; very large = 0.7–0.9; almost perfect = 0.9 and perfect = 1.0 at a significance of *p* < 0.05).

The level of significance was determined at *p* < 0.05, and the statistical analysis of the data was carried out with the SPSS 21.0 software.

## 3. Results

The Shapiro–Wilk-W tests revealed that one part of the data did not complete the requirements of the normal distribution (*p* = 0.021–0.17; *p* < 0.05) (Table 1); therefore, the non-parametric tests were used to calculate correlation between the variables as well, but regression analysis was not applied.

The ICC for all tests were received (ICC = 0.86–0.99; *p* < 0.05) so all tests were considered to be reliable (Table 2).

**Figure 3:**
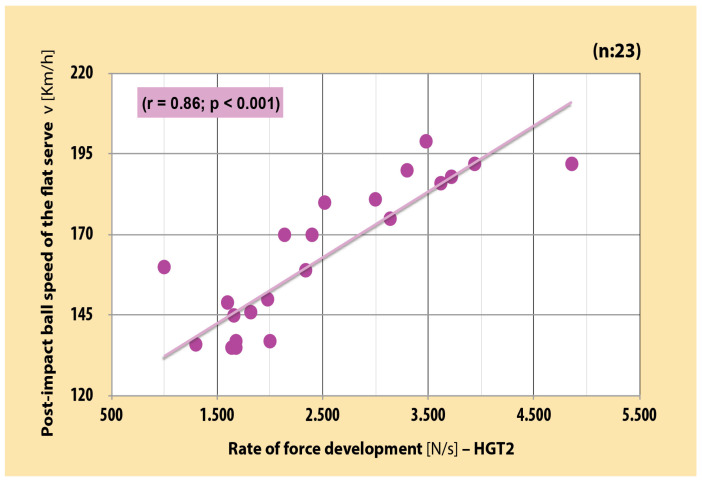
Correlation between RFD of dominant arm in the basic position and PIBS in junior boy tennis players (r = 0.86 [CI, 0.71–0.94]; *p* < 0.001). (self-produced figure). Legend: HGT2—handgrip test 2.

**Figure 4:**
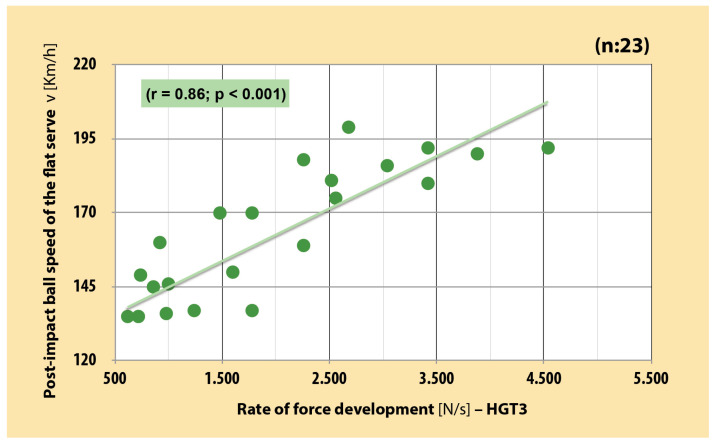
Correlation between RFD of dominant arm in shoulder position and PIBS in junior boy tennis players (r = 0.86 [CI, 0.70–0.94]; *p* < 0.001). (self-produced figure). Legend: HGT3—handgrip test 3.

**Figure 5:**
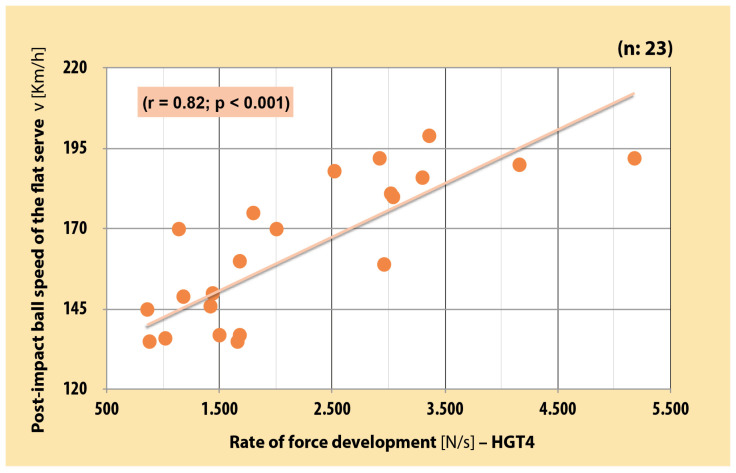
Correlation between RFD of dominant arm in serve position and PIBS in junior boy tennis players (r = 0.82 [CI, 0.63–0.92]; *p* < 0.001). (self-produced figure). Legend: HGT4—handgrip test 4.

**Figure 6:**
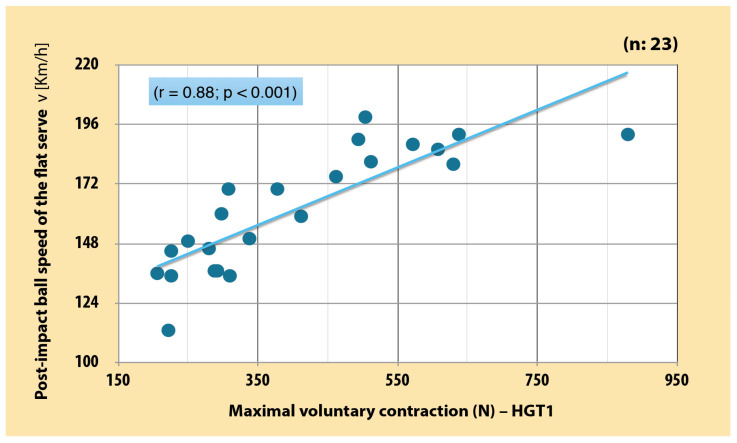
Correlation between MVC of dominant arm in basic position and PIBS in junior boy tennis players (r = 0.88 [CI. 0.73–0.94]; *p* < 0.001). (self-produced figure). Legend: HGT1—handgrip test 1.

There was a very large significant positive correlation between RFD of the dominant arm in each three position and PIBS in junior boy tennis players (r = 0.82–0.86 [CI, 0.63–0.94]; *p* < 0.001) (Figure 3, Figure 4 and Figure 5). As far as MVC is concerned, very large significant positive correlation was also found between MVC of the dominant arm and PIBS (r = 0.88 [CI, 0.73–0.94]; *p* < 0.001) (Figure 6).

## 4. Discussion

In the present research, we hypothesized that RFD of the dominant arm during the handgrip task performed in the three different positions is associated with PIBS, which according to the author’s knowledge is the first study to examine this special topic in elite junior tennis players. The present results demonstrated that there were very large, significant positive correlations between RFD of the dominant arm in each of the three positions and PIBS in junior boy tennis players. Furthermore, it was observed that the MVC of the dominant arm had a very large and significant positive correlation with the PIBS.

### 4.1. Role RFD of Dominant Arm Is Manifested in PIBS in a Flat Serve

Due to modern technology and materials, nowadays the rackets are lighter than the wooden ones [26]. Because of this, a greater racket head speed can be achieved at the contact point that requires the proper ability of the player to manoeuvre the faster racket head. This statement is true especially in the case of flat serve, which is one of the most important and most complex technical elements in junior tennis players, and the execution of which is independent of the opponent’s ball as well [6,27]. In addition, it ensures the highest possible level of movement control of the highest racket head and ball speed achievement [6,28].

The racket in the hand of the player can be modelled as a complex hinged system, in which each segment of the model is connected together by joints of different free-rate and range of movement ability. This complex hinged system moves the racket along the proper path with optimal speed according to a special movement pattern characteristic for the stroke, the aim of which is to reach the proper racket head speed at the contact point (racket and ball collision).

**Figure 7:**
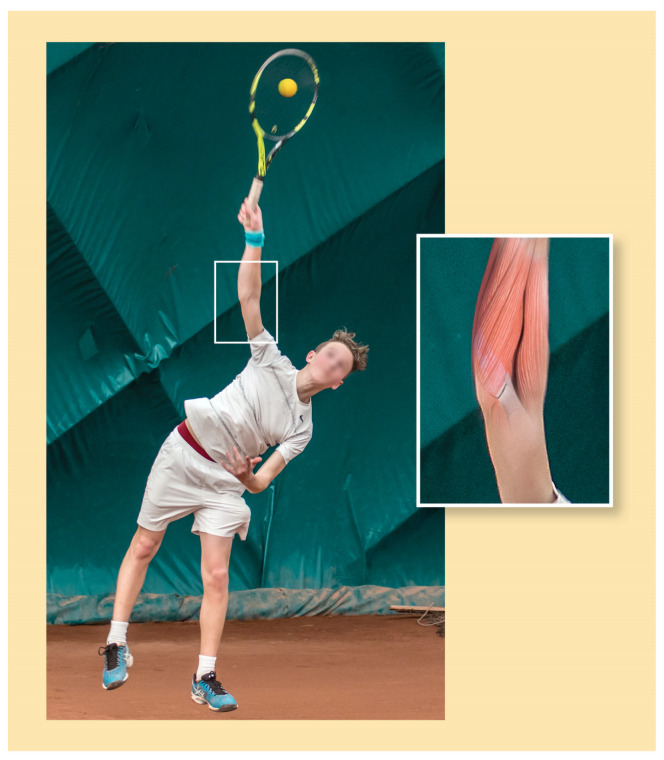
Contact point of flat serve and muscle contraction of flexor muscles in forearm and wrist of the dominant arm. (self-produced figure).

Forearm and wrist movements of the dominant arm are the last elements of a kinetic chain in the flat serve, where energy of the body movement is transferred to the forearm and wrist and is released at the contact point (Figure 7). The contact point is a key element of the flat serve where substantial wrist flexor activities are observed [29,30,31]. However, previously it was mentioned that the proper impulse that changes the momentum of the ball has to be expressed within an extremely short duration of contact time (0.003–0.006 s).

In addition, in the case of a flat serve, the momentum of the free-fall ball point has to be overcome with a higher impulse (mass × speed) of the racket, in contrast to the ground stroke, where the aim is to control the ball arriving from the front on a ballistic path [6]. Furthermore, it should be noted that basic shots (forehand and backhand) are often executed with topspin, especially the forehand shots. Controlling and hitting these incoming topspin balls on a proper target area also present a great challenge to tennis players.

In the contact point, the most optimal force execution is given by the flat serve [29], as the face of the racket is almost in full degree to the horizontal line at the contact point [20]. In addition, if the force impact is not optimal (the direction vector of follow-through does not coincide with the speed vector of the ball’s centre of gravity), thus the plain of the racket is not a suitable place at the contact point, the movement energy of the racket is not transferred properly to the ball, and the post-impact ball speed decreases. Therefore, during the execution of the flat serve, the players try to create a proper (square) racket face and maintain the force on the racquet in the direction of the serve for as long as possible to increase the momentum of the racquet transferred to the ball [28]. It may be that in the case of a flat serve, the quick stabilizing process of the racket’s head at the contact point (Figure 7), in which the rapid force generation of the forearm and wrist muscles of the dominant arm is determinant, may probably aid the more optimal force transfer.

Of course, the player’s level of coordination ability and power, the “heaviness” of the stroke, the elastic characteristics of the racket strings, the internal pressure of the ball, and the actual state of its surface also play a role in the speed of the serve [29]. However, the higher speed of the ball stemming from the string elasticity is only 1 or 2% [32], and all players used new balls uniformly during the testing session. Furthermore, junior boy tennis players who had proper condition and possessed the right serve motion participated in the research. That is why, based on the gained data, the statement seems to be realistic: the RFD of the players’ dominant arm may play a role in the more effective formation of the force transfer between the racket and the ball (at the contact point) that is manifested in the PIBS.

### 4.2. MVC Performance of Dominant Arm

As mentioned earlier, upon investigation of the correlation between the MVC of the dominant arm and the PIBS, the results of different studies were contradictory. In a previous study [10,11,13], a significant correlation was found between MVC of the dominant arm and PIBS in junior tennis players. Correlation between MVC of the dominant arm and PIBS in national groups of players was also found in a study [15]; however, no such significant correlation was observed among professional players. Furthermore, another analysis found [12,14], no correlation between MVC of the dominant arm and PIBS in professional male and intercollegiate tennis players. In the present study, a very large, significant positive correlation was found between the MVC and the PIBS that was in line with another similar study conducted on junior tennis players [10,11,13].

The conclusion can be drawn that MVC is a good general strength indicator of the upper extremities or even of the general muscular strength [33,34]. Furthermore, several studies [35,36] have proven a strong correlation between MVC and RFD in various other sports. In addition, during the proper technical preparation process, the importance of general physical attributes and improved fitness becomes relevant, as they seem to contribute to generating powerful shots [11]. It is important to note that most of the tennis strokes, especially the flat serve, are of power nature, as the aim is to generate force quickly [5]. Furthermore, as mentioned previously, tennis serve does not require maximal grip strength, as suggested by the findings of Lucki and Nicolay [14]. Therefore, the MVC of the dominant arm seems to play a role in the prevention of tennis players’ injuries, such as tennis elbow or glenohumeral joint injury [37]. In addition, MVC measurement is widely used to assess the asymmetry between the dominant and non-dominant arm in tennis players, and age- and gender-related and normative values are available about tennis players [33,38].

### 4.3. Importance of Flat Serve and RFD of Dominant Arm as Well as Limitation of Present Study

International data show that older junior boy tennis players (aged 16–18 years) in the top 1000 of the ITF ranking have on average 72 (49–103) serves per match, a great percentage of which is executed with an average speed of 158 (119–193) km/h flat serve [39], and 66.7 (59.3–74.2) percentage of points is won from the rallies of their first serves [39]. Besides this, junior players have 23–56 matches/year on average at the international level [39] that may be supplemented by a great number of national matches. Furthermore, based on general results, males can serve 8–10% better scores than female tennis players [40]. A previous study [41] has indicated that the serve’s speed and number of aces and points from the first service are significantly lower in younger junior boy tennis players (aged 12–16 years) than for older junior boy players. This observation may be related to the development of technical, physical, and tactical skills and anthropometric characteristics in younger players. However, it is important to mention that serve can be improved and trained throughout a player’s career, from beginner to professional level [40]. In addition, the serve is the most dominant shot in the game, as well as one of the most important shots from a strategic point of view at all levels [11].

That is why it can be supposed that the examined junior tennis players during their 30–60 matches/year execute an outstanding number of flat service/matches. That is why it is not all the same what the PIBS is, as the ability of the players to generate high-speed balls has become one of the fundamentals of a successful competition performance [38]. Therefore, it seems that RFD of the dominant arm is an important variable in tennis, as the head of the racket has to be placed in the optimal position within a fraction of a second in order to ensure a proper contact point, which may probably contribute to optimal racquet-ball collision. Furthermore, steepness of force impact (RFD), its rate of change during a force development time unit, shows a closer relation to sport-specific movement form than the MVC [42].

### 4.4. Limitations and Advantages of the Study

It is important to mention the limitations of this present study. First, the small sample size (*n* = 23), so it would also be useful to carry out the research on a greater number of population. Second, it would be interesting to include junior girl tennis players in order to reveal differences according to gender. It should be noted that the present study was not controlled for variables related to the racquet string tension and the condition of the balls (tiny internal differences can occur during manufacturing of the ball).

Despite these limitations, the obtained results are valuable and add relevant information to the examined topic, and this study may be a good starting point for a further investigation of the relationship between the RFD of the dominant hand and the PIBS in elite tennis players.

## 5. Conclusions

Results of this present study indicated that the RFD during a handgrip task performed with the dominant arm in each three positions is associated with the PIBS in a flat serve in selected junior boy tennis players. Therefore, in our opinion, rapid force generation of the forearm and wrist of the dominant arm at the contact point of the flat serve (performed with maximal force) may probably play a role in the formation of a stable racquet head and in the related PIBS compared to the slower MVC during the handgrip task, which much rather characterizes the general strength conditions of a player. Therefore, measurement of the RFD during a handgrip task with dominant arm is suggested in the testing session of the flat serve of junior boy tennis players.

## Figures and Tables

**Table 1:** Descriptive statistics of different variables in junior boy tennis players (n:23).

Variables	Mean ± SD	Min–Max	Shapiro–Wilk	*p*-Value of Shapiro–Wilk
HGT1(N)	405.82 ± 174.57	206–880	0.89	* 0.02
HGT2 (N/s)	2451.39 ± 997.99	1000–4860	0.92	0.10
HGT3 (N/s)	1963.47 ± 1134.03	620–4540	0.91	0.06
HGT4 (N/s)	2164.60 ± 1146.64	860–5180	0.89	* 0.02
ST v (Km/h)	161.95 ± 24.16	113–199	0.94	0.17

Legend: HGT1—handgrip test 1; HGT1—handgrip test; 2; HGT2—handgrip test 3; HGT3—handgrip test 4; HGT4—handgrip test 4; ST—serve test; (* denotes significant difference at *p* < 0.05).

**Table 2:** Reliability values of selected tests for elite junior tennis players.

Tests	HGT1	HGT2	HGT3	HGT4	ST
ICC (95% CI)	0.98 (0.78–0.99)	0.99 (0.92–0.99)	0.86 (0.54–0.99)	0.99 (0.96–0.99)	0.95 (0.46–0.99)
CV%	0.24	0.25	0.22	0.77	0.25

Legend: HGT1—handgrip test 1; HGT2—handgrip test 2; HGT3—handgrip test 3; HGT4—handgrip test 4; ST—serve test; ICC—intraclass correlation coefficient; CI—confidence interval; CV—coefficient of variation.

## Data Availability

The data presented in this study are available on request from the corresponding author.
